# Transient miR-92a Induction in Intermediate Monocytes (CD14^++^CD16^+^) in Acute Coronary Syndrome (ACS)

**DOI:** 10.3390/ijms27073281

**Published:** 2026-04-04

**Authors:** Lukas Harbaum, Julian Kreutz, Carina Weibler, Gerhild Euler, Michael Malysa, Hartmann Raifer, Bernhard Schieffer, Karsten Grote, Mariana Parahuleva

**Affiliations:** 1 Department of Cardiology, Angiology and Intensive Care Medicine, Philipps-Universität Marburg, Baldingerstrasse, 35043 Marburg, Germany; julian.kreutz@uk-gm.de (J.K.); carina_weibler@yahoo.com (C.W.); malysa@staff.uni-marburg.de (M.M.); bernhard.schieffer@uk-gm.de (B.S.); karsten.grote@staff.uni-marburg.de (K.G.); mariana.parahuleva@prof-parahuleva.de (M.P.); 2 Institute of Physiology, Justus Liebig University, 35392 Giessen, Germany; gerhild.euler@physiologie.med.uni-giessen.de; 3 Flow Cytometry Core Facility, Philipps-Universität Marburg, Baldingerstrasse, 35043 Marburg, Germany; raifer@staff.uni-marburg.de

**Keywords:** miR-92a, monocytes, acute coronary syndrome, inflammation

## Abstract

Intermediate monocytes (CD14^++^CD16^+^), a highly pro-inflammatory subset, are linked to endothelial activation, thrombus formation, and poor outcomes in acute coronary syndrome (ACS), suggesting a role in the transition to plaque vulnerability. MicroRNA-92a (miR-92a) promotes vascular inflammation by repressing the transcription factors Kruppel-like factors (KLFs) 2/4, thereby inducing endothelial dysfunction and increasing leukocyte adhesion. Because both intermediate monocytes and miR-92a contribute to plaque instability, their expression profiles appear relevant in acute ischemia. We investigated whether miR-92a is differentially regulated in monocyte subpopulations in ACS compared to chronic coronary syndrome (CCS). Patients with ACS (STEMI/NSTEMI) undergoing urgent coronary angiography and patients with CCS were enrolled. Blood samples were collected peripherally (T_0P_) and from the culprit coronary artery (T_0C_) during catheterization. Additional peripheral samples were collected 48 h after intervention (T_1_) and at the 3-month follow-up (T_2_). Peripheral blood mononuclear cells (PBMCs) were isolated by Ficoll density-gradient centrifugation. Monocytes were sorted by fluorescence-activated cell sorting (FACS) into classical (CD14^++^CD16^–^), intermediate (CD14^++^CD16^+^), and non-classical (CD14^+^CD16^++^) subsets. MiR-92a expression was measured using real-time PCR and analyzed across predefined time points. In classical and non-classical monocytes, miR-92a levels remained stable throughout the observation period and did not differ between ACS and CCS patients. No spatial expression gradient was observed between intracoronary and peripheral samples at baseline. In contrast, intermediate monocytes in the ACS cohort showed a transient increase in miR-92a expression at T_1_ compared with baseline (T_0p_) and the 3-month follow-up (T_2_). No comparable temporal changes were observed in CCS patients. These findings indicate a temporary alteration of miR-92a expression in intermediate monocytes during the early post-interventional phase following ACS. However, given the exploratory nature of this study and the limited sample size, the biological significance of this observation requires confirmation in larger cohorts.

## 1. Introduction

Coronary heart disease (CHD) remains a leading global cause of morbidity and mortality [1], with its two principal clinical phenotypes—acute coronary syndrome (ACS) and chronic coronary syndrome (CCS)—arising from a shared atherosclerotic history despite their varied manifestations [2]. The distinction between them is largely determined by differences in plaque biology and inflammatory activity, as ACS results from plaque rupture or erosion with superimposed thrombosis and marked systemic inflammation, whereas CCS reflects more stable lesions with comparatively attenuated immune activation [3]. Among the immune cells involved, monocytes serve as key orchestrators of atherosclerotic inflammation and its acute complications [4], with their importance becoming particularly evident when examining the functional heterogeneity of monocyte subsets defined by their characteristic expression patterns of cluster of differentiation (CD) 14 and CD16. Classical monocytes (CD14^++^CD16^–^) are recruited early to the inflamed endothelium, where they exhibit phagocytic functions, while non-classical monocytes (CD14^+^CD16^++^) patrol the endothelial surface, surveying vascular injury and contributing to tissue repair. In contrast, intermediate monocytes (CD14^++^CD16^+^), though in a transitional state, display disproportionately high pro-inflammatory and pro-thrombotic activity [5], with elevated proportions consistently associated with endothelial activation, plaque instability, microvascular obstruction, and adverse cardiovascular events [6,7]. In ACS, monocyte subset distributions shift toward these inflammatory phenotypes, with intracoronary sampling demonstrating rapid recruitment and activation within the culprit vessel [8].

Micro-ribonucleic acids (miRNAs) have emerged as key regulators of post-transcriptional immune and vascular signaling, functioning as short endogenous non-coding RNAs that bind to complementary sequences in target messenger RNAs (mRNA), inducing translational repression or mRNA degradation [9]. This broad regulatory capacity allows individual miRNAs to simultaneously modulate multiple signaling pathways and control key processes such as immune cell activation, endothelial homeostasis, and inflammatory gene expression programs [10]. Within the context of cardiovascular disease, miRNA-mediated regulation has been increasingly recognized as an important mechanistic layer that contributes to vascular dysfunction, plaque formation, and the progression of chronic inflammation by fine-tuning gene expression in endothelial cells and leukocytes, influencing cytokine production, and shaping inflammatory responses at all stages of atherosclerosis [11,12]. Among miRNAs implicated in coronary disease, miR-92a has gained particular attention for its potent effects on endothelial activation by repressing the atheroprotective transcription factors Kruppel-like factors (KLFs) 2 and 4, thereby amplifying nuclear factor kappa-light-chain-enhancer of activated B cells (NF-κB)-driven inflammatory signaling, promoting leukocyte adhesion, and reducing nitric oxide bioavailability [13]. Experimental models have shown that miR-92a becomes upregulated in atheroprone vascular regions and in response to inflammatory stimuli, and that its inhibition attenuates endothelial dysfunction while stabilizing atherosclerotic lesions [14]. Circulating levels of miR-92a have been observed to be altered in patients with CHD, most prominently in those with ACS [15,16], and intracoronary gradients suggest local vascular release [17]. However, circulating measurements alone cannot identify the cellular sources of miR-92a or elucidate its regulation within specific immune cell subsets, leaving several critical questions unanswered: whether miR-92a expression differs across monocyte subpopulations, whether such differences distinguish ACS from CCS, and how miR-92a expression changes after coronary intervention. Because understanding cell-type-specific regulation is essential for linking molecular profiles to monocyte-driven inflammation in vivo, this study integrates detailed immunophenotyping with cell-type-resolved miRNA profiling across peripheral and intracoronary compartments to refine the mechanistic understanding of monocyte activation in CHD and evaluate miR-92a as a potential marker of coronary disease activity.

## 2. Results

A total of 14 patients were enrolled in the study, including 8 with ACS and 6 with CCS. Baseline demographic variables were largely similar between groups (Table 1). The distribution of cardiovascular risk factors, such as arterial hypertension, hyperlipidemia, diabetes mellitus, and nicotine abuse, was similarly balanced. Additional comorbidities like chronic obstructive pulmonary disease (COPD), asthma, peripheral arterial occlusive disease (PAD), chronic kidney disease (CKD), and atrial fibrillation (AF) occurred at comparable rates, indicating a generally similar clinical profile at baseline.

All study patients, irrespective of whether they presented with ACS or CCS, had angiographically high-grade or hemodynamically relevant stenosis, and therefore all subjects underwent percutaneous coronary intervention (PCI) with stent implantation. The number of affected coronary vessels was similar between patients with ACS and CCS (*p* = 0.94). In ACS patients, 2 patients had single-vessel disease (25%) (CCS: 0), 2 patients had two-vessel disease (25%) (CCS: 4 [66.7%]), and three-vessel disease was observed 4 times (50%) (CCS: 2 [33.3%]). The location of the treated vessel [Left anterior descending artery (LAD), Left circumflex artery (LCX) or Right coronary artery (RCA)] did not differ significantly between groups. Patients with ACS required a significantly greater number of stents than those with CCS (ACS median 2.00, IQR 2.00–3.75 vs. CCS median 1.00, IQR 1.00–1.25; *p* = 0.01). All patients received guideline-directed medical therapy based on clinical indications. CCS patients who underwent percutaneous coronary intervention and stent placement were treated with dual antiplatelet therapy consisting of aspirin and clopidogrel. Patients with ACS generally received dual antiplatelet therapy with aspirin and prasugrel. When prasugrel was contraindicated (e.g., advanced age, increased bleeding risk, or relevant medical history), dual antiplatelet therapy with aspirin and clopidogrel was used instead. Patients with a history of atrial fibrillation were subsequently treated with DOACs and clopidogrel.

Periprocedural and in-hospital events were systematically documented. No significant differences were observed between ACS and CCS patients regarding vascular access site bleeding, blood transfusion needs, or the occurrence of significant hematoma. In the ACS group, acute kidney injury affected 25% of patients, with one patient (13%) requiring continuous venovenous hemodialysis and filtration (CVVHDF). Conversely, no cases of acute kidney injury or CVVHDF were reported in the CCS group. One patient with ACS received temporary mechanical circulatory support due to early-stage cardiogenic shock. There were no strokes or emergency coronary artery bypass surgeries in either group. Pneumonia was more common in the ACS group (25% vs. 0%), although this difference was not statistically significant (*p* = 0.47). The length of hospital stay was notably longer for ACS patients compared to CCS patients (median 9.50 days, IQR 7.75–15.25 vs. median 2.00 days, IQR 2.00–3.00; *p* = 0.006).

For each patient, a comprehensive laboratory panel was conducted (Table 2), including markers of cardiac injury and heart failure (CK, CK-MB, high-sensitivity troponin I [hs-TnI], and NT-proBNP), renal and hepatic parameters (creatinine, AST, and ALT), inflammatory markers (leukocyte count and CRP), and lipid profiles (total cholesterol, HDL, and LDL). Significant differences between ACS and CCS patients mainly appeared in markers of myocardial injury and inflammation. As expected, hs-TnI levels were significantly higher in ACS patients (T_0_: *p* = 0.002). Inflammatory activity was also elevated in the ACS group, with significantly increased CRP levels at baseline (T_0_: *p* = 0.028) and follow-up (T_1_: *p* = 0.007), indicating an acute inflammatory response. Additionally, hepatic transaminase was notably higher in ACS patients at baseline (AST T_0_: *p* = 0.003) and T_1_ (*p* = 0.017), suggesting transient hepatic congestion during the acute phase. Importantly, these differences in inflammatory markers and liver enzymes were no longer significant at the later control time point (T_2_). NT-proBNP levels differed significantly between groups at T_1_ (*p* = 0.028). No significant differences were found for creatinine, leukocyte count, CK, CK-MB, ALT, or lipid parameters at any time point.

**Figure 1:**
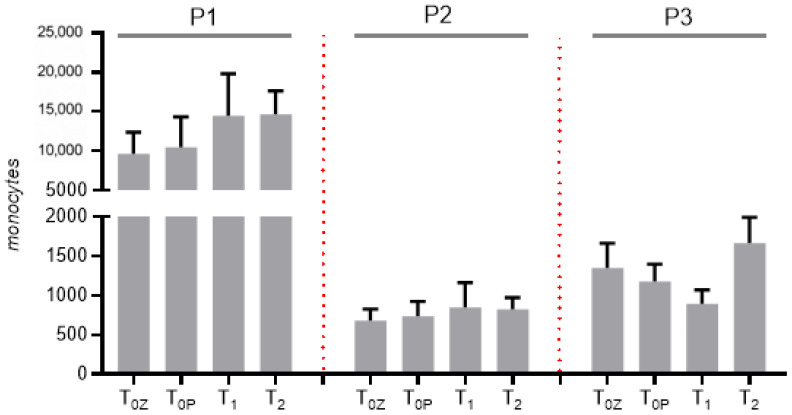
Absolute numbers of sorted monocyte subsets at the respective sampling time points across the entire study cohort. Cell numbers were determined after FACS-based sorting and include samples from both ACS and CCS patients. P1: classical monocytes (CD14^++^ CD16^–^); P2: non-classical monocytes (CD14^+^ CD16^++^); P3: intermediate monocytes (CD14^++^ CD16^+^). T_0c_: time of blood sampling from the coronary vessel; T_0p_: time of blood sampling from peripheral vein during coronary angiography; T_1_: blood sampling 48 h after procedure; T_2_: blood sampling 3 months after procedure. Data are presented as bar graphs showing the mean ± SEM.

For subsequent reverse transcription polymerase chain reaction (RT-PCR) analysis, flow cytometry was performed at all predefined time points: intracoronary baseline (T_0C_), peripheral baseline (T_0P_), 48 h (T_1_), and 3 months (T_2_). First, we analyzed the entire study population regardless of clinical presentation in order to maximize statistical power and identify potential differences in the distribution of monocyte subpopulations. Figure 1 shows, as expected, that classical monocytes clearly outnumbered the other monocyte types.

**Figure 2:**
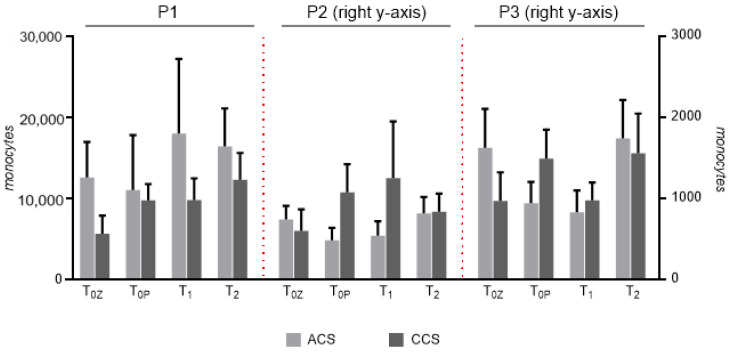
Absolute monocyte counts in the respective populations at the various sampling times in ACS and CCS patients were assessed by FACS analysis. P1: classical monocytes (CD14^++^ CD16^–^); P2: non-classical monocytes (CD14^+^ CD16^++^); P3: intermediate monocytes (CD14^++^ CD16^+^). T_0c_: time of blood sampling from the coronary vessel; T_0p_: time of blood sampling from peripheral vein during coronary angiography; T_1_: blood sampling 48 h after procedure; T_2_: blood sampling 3 months after procedure. Data are presented as bar graphs showing the mean ± SEM.

The distributions across all three subpopulations remained consistent throughout the observation period. One-way ANOVA showed no significant changes within individuals. Stratification by clinical presentation (ACS vs. CCS; Figure 2) also revealed no significant group-dependent differences at any time point. Minor numerical variations were inconsistent and did not reach statistical significance. We further observed no differences between peripheral and coronary blood sampling at baseline intracoronary (T_0c_) and peripheral (T_0P_), either in the entire study population or by clinical presentation. Overall, monocyte subset profiles stayed stable across the acute, subacute, and chronic phases and were similar between patients with ACS and CCS.

**Figure 3:**
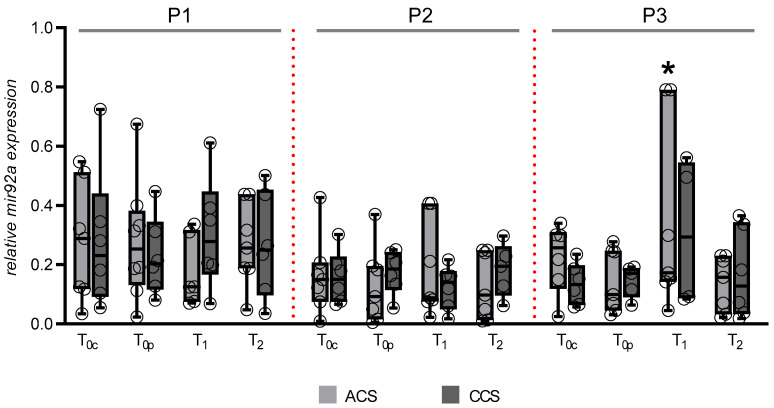
Relative expression of miRNA-92a in the respective monocyte population at the various sampling times in ACS and CCS patients was determined by RT-PCR. P1: classical monocytes (CD14^++^ CD16^–^); P2: non-classical monocytes (CD14^+^ CD16^++^); P3: intermediate monocytes (CD14^++^ CD16^+^). T_0c_: time of blood sampling from the coronary vessel; T_0p_: time of blood sampling from peripheral vein during coronary angiography; T_1_: blood sampling 48 h after procedure; T_2_: blood sampling 3 months after procedure. Data are presented as box-and-whisker plots with individual data points overlaid. Boxes indicate the median and interquartile range; * *p* < 0.05 vs. T_0p_ (ACS) and T_2_ (ACS).

Subsequently, miR-92a expression in monocyte subpopulations was analyzed using RT-PCR to identify subpopulation-specific regulatory patterns at different study time points. Quantitative analysis showed stable levels of miR-92a in both classical and non-classical monocytes across all predefined time points (Figure 3), with no significant changes over time. In contrast, intermediate monocytes exhibited a transient increase in miR-92a expression in the ACS group. miR-92a levels rose from 0.145 ± 0.107 at T_0p_ to 0.342 ± 0.315 at T_1_, then decreased to 0.136 ± 0.093 at T_2_. Post hoc analysis revealed significant differences between T_0p_ and T_1_ (*p* = 0.034) and between T_1_ and T_2_ (*p* = 0.028). No similar temporal changes were found in the CCS group. There were no significant differences in miR-92a expression between ACS and CCS patients at baseline (T_0C_ or T_0P_) or at the 3-month follow-up (T_2_). Similarly, miR-92a expression in the CCS group remained stable across all sampling points. Overall, these results suggest a transient increase in miR-92a expression in intermediate monocytes during the early post-interventional phase in ACS patients, although this association did not remain statistically significant after Benjamini–Hochberg correction for multiple testing.

## 3. Discussion

This study demonstrates a transient increase in miR-92a expression in intermediate monocytes during the early post-interventional phase of ACS. These findings indicate that post-transcriptional regulatory mechanisms involving microRNAs are dynamically modulated in circulating immune cells during the inflammatory response following myocardial ischemia.

Intermediate monocytes have repeatedly been identified as a key leukocyte subset involved in the inflammatory response to coronary plaque disruption. Beyond their phenotypic distinctiveness, high-dimensional profiling studies have revealed that they upregulate gene programs related to antigen presentation, chemotaxis, and cytokine signaling while exhibiting increased adhesive and prothrombotic behavior [18,19,20]. Their kinetics are well characterized, as following myocardial ischemia, intermediate monocyte levels peak within 24–72 h, coinciding with the transition from early innate immune activation to the initiation of reparative processes [6].

The timing of miR-92a induction seen in this study aligns with the established period of intermediate monocyte activation. This timing suggests that miRNA-based regulatory mechanisms might influence the functional state of this monocyte subset during the early inflammatory phase after ACS. However, the current study does not determine the downstream functional effects of miR-92a expression in these cells.

MiR-92a has previously been implicated in vascular inflammation and endothelial activation in experimental and translational studies. In endothelial cells, increased miR-92a expression has been associated with enhanced leukocyte adhesion through upregulation of vascular adhesion molecule (VCAM)-1, intracellular adhesion molecule (ICAM)-1, and endothelial (E)-selectin, while reducing nitric oxide bioavailability via repression of KLF2 and KLF4 [21,22,23]. Experimental models have consistently demonstrated that miR-92a responds to pro-inflammatory and biomechanical stimuli—such as disturbed flow, oxidative stress and cytokine exposure—thereby amplifying vascular inflammation, and that its inhibition reduces endothelial activation, improves vascular regeneration, and weakens plaque formation in atherosclerosis [14]. While these observations highlight the broader biological relevance of miR-92a in cardiovascular pathology, the current data do not directly establish comparable regulatory mechanisms within intermediate monocytes but may draw attention to miR-92a’s relevance not only in endothelial signaling but also in circulating immune cells during the inflammatory surge following ACS.

However, clinical studies measuring circulating miR-92a levels have reported heterogeneity in timing, magnitude, and clinical correlations, ranging from associations with infarct size and troponin release to observations of transcoronary gradients suggestive of localized vascular release [16,24]. One potential explanation for this variability is that circulating miRNA measurements reflect composite signals from multiple tissues and cell types. The present analysis could provide cell-type-resolved data demonstrating that miR-92a induction might not be uniformly distributed across the monocyte compartment but occurs predominantly in intermediate monocytes during a defined temporal window following the ischemic event. This observation may help explain the variability observed in plasma-based studies.

Notably, the absence of miR-92a changes in both classical and non-classical monocytes is evident despite their contribution to post-ischemic immune responses, as their transcriptional and functional programs differ substantially from those of intermediate monocytes and are governed by distinct regulatory networks [19,25]. The finding that miR-92a expression is induced only in intermediate monocytes underscores the heterogeneity of monocyte involvement in ACS.

While miR-92a expression did not differ between ACS and CCS in cross-sectional comparisons, a significant time-dependent increase was observed only in ACS but not in CCS. This further supports the concept that acute plaque destabilization is accompanied by a qualitatively different inflammatory milieu than that present in stable coronary disease. Previous clinical studies have shown that patients with ACS exhibit a markedly altered systemic cytokine profile compared with stable CCS, reflecting heightened immune activation and acute inflammatory signaling [26]. Monocytic activation markers such as tissue factor or CD14 are also more strongly induced in unstable coronary syndromes than in stable angina, underscoring a functionally different prothrombotic-inflammatory cell response [27,28]. Consistent with this concept, patients with ACS in the present study exhibited significantly higher levels of inflammatory and myocardial injury biomarkers at time point T_1_ compared with patients with CCS, including C-reactive protein (CRP), troponin, and NT-proBNP. These findings confirm that the observed miR-92a induction in intermediate monocytes occurs in the context of a systemic inflammatory and myocardial stress response characteristic of the acute ischemic setting. While the present analysis does not establish a direct mechanistic link between miR-92a expression and these biomarkers, their concurrent elevation supports the interpretation that the miR-92a signal reflects immune activation occurring during the early phase of ACS.

Treatment-related differences should also be considered when interpreting the observed molecular patterns. Although all patients in the present study underwent PCI with stent implantation, antiplatelet therapy differed between groups according to clinical indication. This may be relevant, as P2Y12 inhibition has been shown to attenuate platelet-induced proinflammatory monocyte programming, including transcriptional responses [29], and prasugrel has been reported to reduce platelet–monocyte aggregate formation more effectively than clopidogrel [30]. Therefore, part of the observed differences between ACS and CCS may reflect treatment-associated effects in addition to disease-specific inflammatory signaling.

Although the proportions of the other monocyte subsets were similar across groups, the molecular activation patterns diverged, highlighting that functional changes in monocytes may precede or occur independently of numerical expansion. This is consistent with transcriptomic studies showing that monocytes in ACS exhibit distinct inflammatory signatures even without discernible shifts in subset distribution [31,32].

Interest in leukocyte-derived microRNAs as markers of inflammatory activation and cardiovascular risk has grown in recent years. Subset-specific profiling may provide greater mechanistic insight than circulating miRNA measurements alone, which reflect signals from multiple cellular sources. In this context, the transient increase in miR-92a in intermediate monocytes observed in this study may reflect early immune activation following myocardial ischemia. However, larger studies are required to validate these findings and to determine whether miR-92a expression in monocyte subsets is linked to specific functional pathways or clinical outcomes, ideally with sufficient statistical power to allow robust correction for multiple testing.

## 4. Materials and Methods

### 4.1. Study Population and Study Design

**Figure 4:**
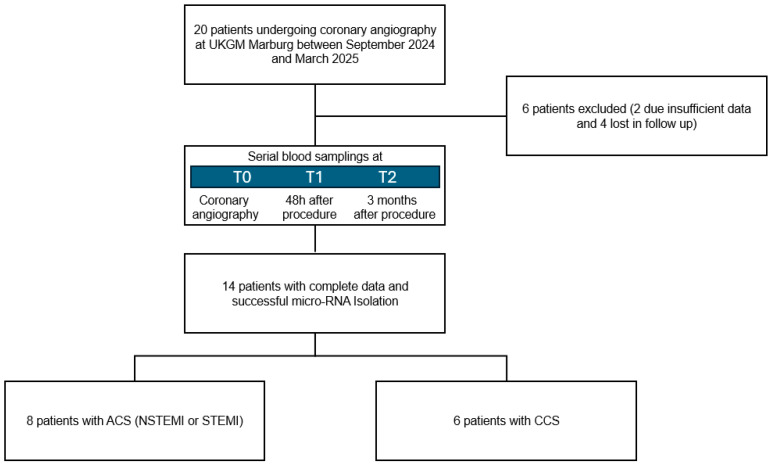
Flow chart of the study population.

This monocentric study enrolled patients undergoing coronary angiography in accordance with guidelines between September 2024 and March 2025. Two cohorts were included (Figure 4): patients with ACS and those with CCS. Eligible participants were adults aged 18–85 with at least one significant coronary stenosis and the capacity to provide informed consent. Patients were excluded if they had experienced a recent myocardial infarction or percutaneous coronary intervention, had active malignancy, uncontrolled endocrine or cardiovascular disease, chronic inflammatory or infectious conditions, central organ dysfunction, psychiatric disorders that would affect their ability to participate, were pregnant, had previously undergone coronary artery bypass surgery, had undergone major surgery recently, had experienced an acute cerebrovascular event recently, or had an inadequate baseline hemoglobin level. Patients could withdraw from the study at any time, and participation could also be discontinued in the event of death or a ≥2.0 g/dL decline in hemoglobin. All patients underwent a standardized clinical assessment including a medical history, cardiovascular risk profiling, a physical examination, an electrocardiogram, echocardiography, and routine laboratory testing. Blood samples were obtained three times. At T_0_, intracoronary blood was obtained via a selectively engaged guiding catheter positioned at the coronary ostium of the culprit vessel during angiography, and a peripheral vein sample was obtained. Additional venous samples were collected 48 h after the procedure (T_1_) and again after three months (T_2_).

This study was conducted in accordance with the Declaration of Helsinki and was approved by the local institutional ethics committee (approval number 59/21; 31 May 2021). All participants provided written informed consent before enrolment, and all clinical procedures followed standard care guidelines, regardless of study participation.

### 4.2. Laboratory Parameters

Routine laboratory parameters were measured at the central clinical laboratory of the University Hospital Giessen and Marburg (UKGM), Marburg, using standardized diagnostic procedures. Blood samples were collected as part of routine care and processed following institutional protocols. CRP concentrations were determined with an automated immunoturbidimetric assay, and NT-proBNP levels were measured using an electrochemiluminescence immunoassay on certified automated analyzers. Additional laboratory parameters were analyzed with standard automated clinical chemistry or immunoassay platforms. The measured concentrations are summarized in Table 2.

### 4.3. Blood Collection and Cryopreservation

During each blood sampling, approximately 40 mL (4 × 10 mL) of ethylenediaminetetraacetic acid (EDTA) whole blood (Sarstedt, Nuembrecht, Germany) was collected. Intracoronary blood was obtained at baseline via a standard 6F catheter after selective engagement of the culprit coronary ostium, sampling from the continuously perfused coronary circulation (prior to PCI). Density gradient centrifugation with Pancoll human (PAN-Biotech, Aidenbach, Germany) was performed on each 10 mL samples of EDTA blood to isolate peripheral blood mononuclear cells (PBMCs, 4 aliquots a ~1 × 10^7^ cells). The interphase was washed in phosphate-buffered saline (PBS) and then transferred to the Cryo-SFM freezing medium (Promocell, Heidelberg, Germany). The samples were gradually frozen using Mr. Frosty™ Freezing Containers (Thermo Scientific, Waltham, MA, USA) and cryopreserved in liquid nitrogen until further processing.

### 4.4. Flow Cytometry and Cell Sorting

For flow cytometric analysis and cell sorting of monocyte subpopulations, one aliquot of frozen peripheral blood mononuclear cells (PBMCs) were thawed, washed, and incubated with fluorochrome-conjugated antibodies (BD Biosciences, San Jose, CA, USA). The antibody panel included CD2 (PE, clone RPA-2.10; T cells), CD15 (PE, clone HI98; granulocytes), CD19 (PE, clone HIB19; B cells), CD56 (PE, clone MY31; NK cells), and CD335 (PE, clone 9E2; NK cells) as lineage markers, as well as CD14 (APC, clone M5E2; monocytes), CD16 (PE-Cy7, clone 3G8; monocytes), and HLA-DR (FITC, clone TU36; antigen-presenting cells). Importantly, CD14 and CD16 were not included in the lineage cocktail in order to allow subsequent identification of monocyte subsets based on CD14/CD16 expression. Dead cells were excluded using 4′,6-diamidino-2-phenylindole (DAPI; Sigma-Aldrich, Munich, Germany).

After staining, cells were filtered through a 70 µm cell strainer and sorted using a MoFlo Astrios Cell Sorter (Beckman Coulter, Indianapolis, IN, USA) at the Flow Cytometry Core Facility Marburg.

**Figure 5:**
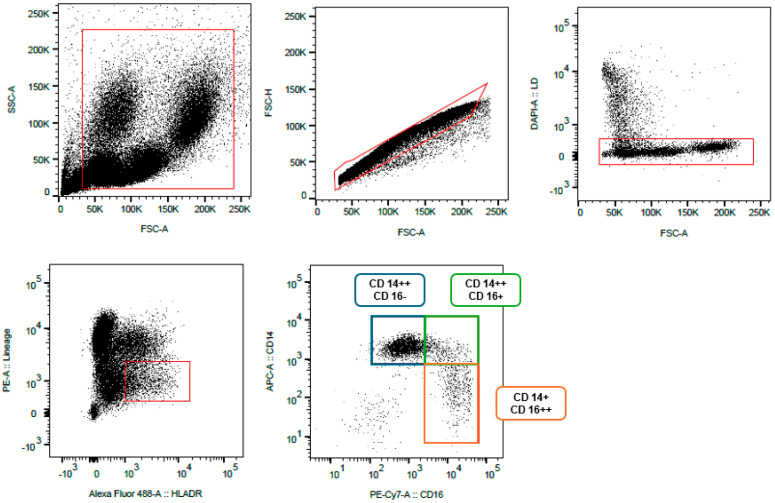
Gating strategy and example of a FACS. P1: classical monocytes (CD14^++^ CD16^–^); P2: non-classical monocytes (CD14^+^ CD16^++^); P3: intermediate monocytes (CD14^++^ CD16^+^); red boxes represent the positively selected cells.

The gating strategy is illustrated in Figure 5. First, mononuclear leukocytes were identified based on forward and side scatter characteristics (FSC-A/SSC-A). Doublets were excluded using FSC-H/FSC-A, and viable cells were selected by excluding DAPI^+^ events. Lineage-positive cells (CD2, CD15, CD19, CD56, CD335) were subsequently excluded, and HLA-DR^+^ cells were selected. Within the Lineage^−^ HLA-DR^+^ population, monocytes were identified and subdivided according to CD14 and CD16 expression into three subsets:

- P1: classical monocytes (CD14^++^ CD16^–^);
- P2: non-classical monocytes (CD14^+^ CD16^++^);
- P3: intermediate monocytes (CD14^++^ CD16^+^).

Sorted cells were directly collected into the RNA-Solv reagent (Omega Bio-tek, Norcross, GA, USA) for immediate RNA stabilization and subsequently stored at −80 °C until RNA isolation. Post-sort purity assessment in independent cryopreserved samples confirmed the robustness of the applied gating strategy. The purity was 97.2% for classical, 92.5% for non-classical, and 97.8% for intermediate monocytes.

### 4.5. RNA Isolation and Quantitative RT-PCR

To obtain total RNA, 200 µL of chloroform (Carl Roth, Karlsruhe, Germany) was added per milliliter of lysis reagent. Subsequently, the samples were centrifuged, and the supernatant was collected. Isopropanol (Carl Roth) was used for RNA precipitation, and GlycoBlue (Thermo Fisher Scientific, Waltham, MA, USA) was used to improve visibility of the RNA pellet. After several washing steps with 75% ice-cold ethanol (Carl Roth), the RNA was air-dried. The pellet was dissolved in RNase-free water and stored at −80 °C. cDNA synthesis and amplification were performed using the TaqMan^TM^ MicroRNA Reverse Transcription Kit (Thermo Fisher Scientific) according to the manufacturer’s protocol. RT-PCR was performed using the TaqMan^TM^ Fast Advanced Master Mix (Applied Biosystems, Darmstadt, Germany) on a StepOnePlus RT-PCR System (Applied Biosystems) in 96-well plates. Probe and Primers, both for cDNA synthesis and RT-PCR, were obtained from the TaqMan^TM^ MicroRNA Assay (Thermo Fisher Scientific). MiRNA-expression was considered detectable if the threshold cycle (Ct value) was less than 35.

### 4.6. MiRNA Normalization Strategy and RT-PCR Parameters

For normalization, RNA RNU48 was used as the endogenous reference gene. To verify RNU48′s suitability as a reference across the analyzed monocyte subsets, disease groups, and sampling time points, the stability of RNU48 Ct values was retrospectively evaluated using the BestKeeper algorithm. BestKeeper assesses candidate reference genes based on Ct value variation across all samples, considering genes with a standard deviation (SD) of Ct values < 1 as stable and suitable housekeeping genes [33]. In this dataset, RNU48 showed a mean Ct value of 28.78 with a standard deviation of 0.89, indicating stable expression across all samples and conditions, supporting its use for normalization. The choice of RNU48 as an endogenous reference gene was based on prior work by Xu et al., who used RNU48 for normalization of miRNA expression in bone marrow–derived mononuclear cells from cardiovascular disease patients [34]. RT-PCR was performed to measure miR-92a levels in sorted monocyte subsets. All reactions were run in technical duplicates for both miR-92a and the endogenous reference gene RNU48, with mean Ct values used for further analysis. Across all samples, cell populations, and time points, the mean Ct value for miR-92a was 29.72 ± 1.90 (SD). A standard curve was not included in the RT-PCR runs because the study employed a relative quantification method (2^−^ΔCt) using validated TaqMan assays [35]. Samples with Ct values near the assay detection limit (around Ct ≈ 35) were included in the analysis.

### 4.7. Statistical Analysis

Statistical analyses were conducted using GraphPad Prism (version 9). Due to the exploratory nature of the study and the small sample size, non-parametric tests were mainly used. Continuous variables are shown as median and interquartile range (IQR). The Mann–Whitney U test was used for comparisons between two independent groups, while the Wilcoxon matched-pairs signed-rank test analyzed paired comparisons. The Friedman test followed by Dunn’s multiple comparisons test was used to analyze differences across more than two related time points.

Categorical variables are presented as counts and percentages and were compared using the Chi-squared test or Fisher’s exact test, as appropriate.

One-way and two-way ANOVAs were applied to analyze monocyte subsets and RT-PCR-derived miR-92a expression, evaluating group differences (ACS vs. CCS), temporal changes across sampling points, and interaction effects. Where ANOVA indicated statistical significance, Fisher’s LSD post hoc test or the Kruskal–Wallis test with Dunn’s multiple comparisons test was applied. Benjamini–Hochberg FDR was applied for multiple testing correction. Repeated measures ANOVA was used where appropriate to account for intra-individual longitudinal sampling. Data visualization and graphical outputs were generated in GraphPad Prism. All statistical tests were two-sided, and *p*-values < 0.05 were considered statistically significant. Given the exploratory nature of this pilot study, results should be interpreted accordingly.

## 5. Conclusions

This study demonstrates a transient increase in miR-92a expression in intermediate monocytes during the early post-interventional phase of acute coronary syndrome. The time-dependent induction observed exclusively in ACS, but not in CCS, suggests that miR-92a may reflect dynamic immune activation during the acute ischemic response. These findings highlight the potential value of cell-type-specific miRNA profiling for improving the understanding of inflammatory processes in cardiovascular disease. Further studies are required to clarify the functional role of miR-92a in monocyte subsets and its potential relevance for inflammatory risk stratification.

## Figures and Tables

**Table 1:** Baseline characteristics of the study participants.

	ACS (n = 8)	CCS (n = 6)	*p*-Value
Age (in years)	68.0 (51–78)	67.0 (61–71)	0.95
Sex (male)	7 (88%)	5 (83%)	0.94
Obesity (BMI > 30)	2 (25%)	0 (0%)	0.47
Arterial hypertension	8 (100%)	4 (67%)	0.16
Hyperlipidemia	1 (13%)	1 (17%)	0.94
Nicotine abuse	4 (50%)	5 (83%)	0.30
COPD/asthma	1 (13%)	1 (17%)	0.94
PAD	2 (25%)	1 (17%)	0.94
Diabetes mellitus	3 (38%)	1 (17%)	0.58
CKD	1 (13%)	1 (17%)	0.94
Atrial fibrillation	3 (38%)	1 (17%)	0.58
LVEF (in %)	55.0 (45–60)	60.0 (50–60)	0.76
Aortic valve stenosis	1 (13%)	1 (17%)	0.94
Aortic valve insufficiency	1 (13%)	0 (0%)	0.94
Mitral valve insufficiency	2 (25%)	1 (17%)	0.94
Tricuspid valve insufficiency	1 (13%)	2 (33%)	0.53

ACS: acute coronary syndrome; CCS: chronic coronary syndrome; CKD: chronic kidney disease; COPD: chronic obstructive pulmonary disease; PAD: peripheral artery disease; LVEF: left ventricular ejection fraction.

**Table 2:** Laboratory values (median (IQR)) of patients in the ACS and CCS cohorts.

	ACS T_0_	CCS T_0_	*p*-Value	ACS T_1_	CCS T_1_	*p*-Value	ACS T_2_	CCS T_2_	*p*-Value
CK [in U/L]	459 (95–1266)	101 (70–119)	0.108	457 (74–636)	77 (64–89)	0.181	97 (47–143)	134 (82–157)	0.414
CK-MB [in U/L]	17 (0–154)	0 (0–0)	0.142	11 (0–43)	0 (0–0)	0.142	0 (0–0)	0 (0–0)	0.662
hs-TnI [in ng/L]	5211 (2225–49,037)	7 (6–11)	0.001	8156 (2871–18,356)	22 (15–29)	0.013	10 (8–17)	6 (6–6)	0.043
Creatinine [in umol/L]	1.07 (0.95–1.15)	0.92 (0.69–1.35)	0.755	1.10 (0.99–1.34)	0.99 (0.85–1.28)	0.755	1.03 (0.98–1.16)	1.02 (0.87–1.54)	0.950
Leukocytes [in G/L]	11.06 (5.40–15.05)	5.60 (5.25–5.91)	0.142	9.88 (7.46–10.75)	7.08 (6.40–10.20)	0.491	7.88 (6.87–10.19)	7.29 (4.63–8.47)	0.345
CRP [in mg/L]	8.0 (3.1–23.0)	1.7 (1.1–2.8)	0.029	21.7 (12.8–96.3)	3.7 (2.2–4.6)	0.005	1.0 (0.7–1.2)	1.0 (0.8–2.1)	0.755
NT-proBNP [in pg/mL]	847 (357–3561)	82 (52–944)	0.081	888 (555–14,603)	95 (59–362)	0.029	252 (170–1647)	121 (31–152)	0.142
Cholesterol [in mmol/L]	163 (128–185)	140 (113–172)	0.345	181 (133–227)	144 (134–195)	0.414	148 (124–165)	152 (132–179)	0.491
LDL [in mmol/L]	106 (77–125)	80 (63–97)	0.228	130 (97–149)	88 (67–101)	0.181	79 (70–92)	79 (61–103)	0.852
HDL [in mmol/L]	38 (35–45)	38 (34–56)	0.755	44 (34–48)	38 (35–60)	0.950	46 (38–58)	39 (36–66)	0.573
AST [in U/L]	56 (39–215)	20 (18–23)	0.001	58 (27–153)	21 (20–23)	0.013	26 (23–31)	25 (24–26)	0.950
ALT [in U/L]	35 (27–53)	20 (17–23)	0.081	34 (22–29)	26 (21–26)	0.282	27 (16–38)	25 (11–30)	0.491

CK: Creatine kinase; CK-MB: Creatine kinase-MB isoenzyme; hs-TnI: high-sensitivity cardiac troponin I; CRP: C-reactive protein; LDL: low-density lipoprotein; HDL: high-density lipoprotein; AST: Aspartate aminotransferase; ALT: Alanine aminotransferase; T_0_: time of blood sampling during coronary angiography; T_1_: blood sampling 48 h after procedure; T_2_: blood sampling 3 months after procedure; underlined values indicate significant comparisons.

## Data Availability

The original contributions presented in this study are included in the article. Further inquiries can be directed to the corresponding author.
